# Subcutaneous stretching enlarges adjacent vertebral artery instantly in patients with cervicogenic dizziness: Two case reports

**DOI:** 10.1097/MD.0000000000032643

**Published:** 2023-02-03

**Authors:** Qingtao He, Huiyi Huang, Hongyu Liang, Li-Wei Chou, Zhonghua Fu

**Affiliations:** a Guangdong Second Traditional Chinese Medicine Hospital, Guangzhou, Guangdong, China; b Clinical Medical College of Acupuncture & Moxibustion and Rehabilitation, Guangzhou University of Chinese Medicine, Guangzhou, Guangdong, China; c Department of Physical Therapy and Graduate Institute of Rehabilitation Science, China Medical University, Taichung, Taiwan, China; d Department of Physical Medicine and Rehabilitation, China Medical University Hospital, Taichung, Taiwan, China; e Department of Physical Medicine and Rehabilitation, Asia University Hospital, Asia University, Taichung, Taiwan, China; f The Institute of Fu’s Subcutaneous Needling, Beijing University of Chinese Medicine, Beijing, China.

**Keywords:** blood flow volume, cervicogenic dizziness (CGD), Fu's subcutaneous needling (FSN), subcutaneous stretching, vertebral artery (VA)

## Abstract

Fu's subcutaneous needling (FSN) is effective for cervicogenic dizziness (CGD), which is often a result of vascular problems. Here, we attribute the positive treatment effect of FSN for CGD to improvements in vascular problems.

**Patient concern:**

Two patients were experiencing low quality of life due to reproducible dizziness.

**Diagnosis:**

Two patients with cervical spine disorder, presented with neck pain and reproducible dizziness. Other causes of dizziness were excluded.

**Interventions:**

Case 1 received 1 session of FSN treatment, while case 2 received 3 sessions of FSN treatment in a month.

**Outcomes:**

The dizziness and neck pain experienced by both patients instantly improved significantly after FSN treatment, and the luminal diameter of the vertebral artery (VA) measured by carotid and VA ultrasound enlarged simultaneously up to 1.29-fold and 1.09-fold for both cases. According to the Hagen–Poiseuille equation, the blood flow volume increased 2.77-fold and 1.43-fold, respectively. Case 2 recovered from CGD with 1.19-fold VA luminal diameter increment and about 2.01-fold increase of blood flow volume in a month.

**Lessons:**

Subcutaneous stretching provides a safe, convenient and immediate solution to CGD, and supports the diagnosis and treatment of CGD under carotid and VA ultrasound. This study suggests that stretching subcutaneously can influence adjacent VA, which may also help improve some cerebrovascular diseases.

## 1. Introduction

Dizziness is a common symptom that arises from various disorders, and contributes to 10 million ambulatory visits every year in the US.^[1]^ One type of dizziness, known as cervicogenic dizziness (CGD), is diagnosed when a disorder or lesion of cervical spine is known to be able to cause dizziness, and other causes of dizziness including vestibular, central nervous system, and psychosomatic etiologies are excluded. Clinical diagnostic tests such as cervical torsion test, cervical joint position error, or posturography are necessary in assisting the CGD diagnosis.^[2]^

Although the pathophysiology of CGD is not fully understood, K. Devaraja regarded vascular causes, such as Bow Hunter syndrome, as one of CGD’s attributions.^[3]^ Bow Hunter syndrome is caused by a rotational compression of the vertebral artery (VA), causing vertebrobasilar insufficiency and sometimes stroke.^[4]^ VAs may be compressed by a range of cervical conditions such as osteophytes, herniated discs, spondylosis, or tendinous bands, and present with symptoms of cerebral hypoperfusion ranging from dizziness to posterior circulation stroke.^[5]^ Therefore, CGD is likely related to the hemodynamics of the VAs and should be given due attention for its possibly severe symptoms.

To observe the VA blood flow, we applied the carotid and VA (CVA) ultrasound, which is increasingly used in the diagnosis and treatment evaluation of cerebrovascular diseases. Surprisingly, several recent clinical practices of Fu's subcutaneous needling (FSN), an innovative acupuncture therapy for stretching subcutaneous tissues of the neck to treat dizziness, showed effective outcomes in the instant enlargement of the VA diameter, as measured by CVA ultrasound. Herein, we introduced 2 study cases with complete clinical data.

## 2. Case presentation

### 2.1. Case 1

A 27-year-old female patient complained of a 5-year history of recurrent dizziness. Her dizziness was described as head heaviness, but not having the sensation of intense spinning that is often associated with benign paroxysmal positional vertigo, and was aggravated with neck movements and neck pain. She had blurred vision and no symptoms of spontaneous nystagmus, tinnitus, or hearing loss. X-ray examination of her cervical spine showed that her cervical spine appears straight (see Figure S1, Supplemental Digital Content, http://links.lww.com/MD/I321, which demonstrates the cervical spine X-ray examination). All her symptoms were partly relieved with adequate rest but relapsed frequently. This patient arrived at the acupuncture treatment room of the Guangdong Second Traditional Chinese Medicine Hospital for FSN treatment on September 17, 2021. To exclude other causes of dizziness, blood pressure was measured in supine and standing position to exclude orthostatic hypotension, and showed normal blood pressure. Vestibular diseases were excluded by the head impulse test, nystagmus evaluation, test of skew, Dix–Hallpike, and supine roll test maneuver, all of which showed normal vestibular function. Targeted neurologic examination and gait and balance testing were normal, which excluded cerebrovascular diseases and vestibulo-spinal or cerebellar lesion. Based on the symptoms and x-ray examination, the patient was diagnosed with CGD, although her CGD diagnostic test results were negative.

**Figure 1.**
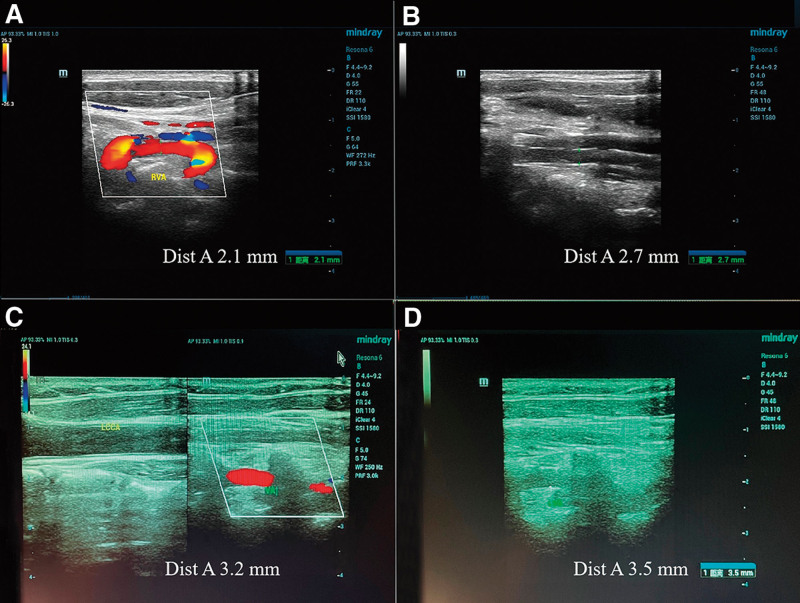
Carotid and vertebral artery ultrasound of 2 cases before and after intervention. Right vertebral artery (V2 segment) luminal diameter was (A) 2.1 mm before, and (B) 2.7 mm after intervention in Case 1. The same outcomes of left vertebral artery in Case 2 were (C) 3.2 mm before, and (D) 3.5 mm after intervention. Dist A = luminal diameter of vertebral artery.

Luminal diameter of the VA passing through the fifth and sixth cervical foramen (V2 segment) was measured immediately before and after intervention, using CVA ultrasound in the same position. Dizziness and neck pain intensities were measured with a numerical rating scale (NRS) of 0 to 10 (1–3: mild, 4–6: moderate, and 7–10: severe). NRS dizziness intensity of the patient was 6, while neck pain intensity was 4, and the luminal diameter was 2.1 mm without evidence of atherosclerosis (Fig. 1A).

**Figure 2.**
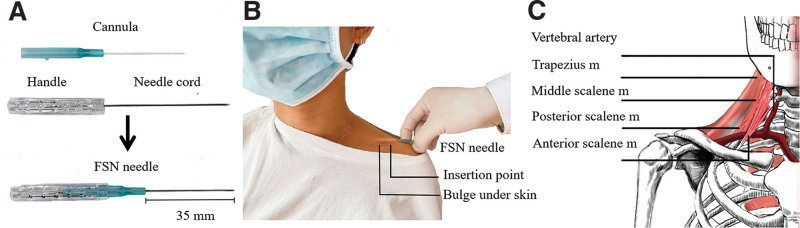
Structure of FSN needle and manipulation of FSN treatment. (A) FSN needle: designed and patented in China (patent number: CN97114318, Nanjing FSN Medical Appliances Co, China). (B) Swaying movement and the insertion point was located at the medial margin of the lateral end of the ipsilateral clavicle. (C) The anatomical position of vertebral artery and scalene muscles. FSN = Fu's subcutaneous needling, m = muscle.

The practitioner first palpated the neck muscles with at least 1 myofascial trigger point (MTrP), which felt stiff and exhibited reduced elasticity.^[6]^ After palpation, MTrPs were found in the scalene muscles of the patient. The FSN needle (Fig. 2A) was then inserted in the medial margin of the lateral end of the ipsilateral clavicle (Fig. 2B and C), and swaying movement of the needle was performed for 1 minute. During the movement, the needle was swayed horizontally from side to side at the angle of about 30° in the subcutaneous layer for about 100 times. The FSN needle stretches the subcutaneous layer, which lies closest to the muscular layer, to stimulate the muscular layer.^[7]^ FSN treatment has been shown to have efficacy in several musculoskeletal disorders.^[8,9]^

Immediately after FSN intervention, a reexamination of the VA using the CVA ultrasound revealed that the luminal diameter of the right VA was 2.7 mm (Fig. 1B), which was 0.6 mm wider. The maximum blood flow velocity (BFV) increased from 50 cm/s to 74 cm/s. NRS dizziness and neck pain intensities reduced to 1 and 2 respectively (Table 1). Simultaneously, the patient reported that her muscle soreness and dizziness disappeared instantly.

**Table 1:** Outcome assessment of 2 cases before and after intervention.

Outcome assessment	First treatment of Case 1			First treatment session of Case 2			Second treatment session of Case 2			Third treatment session of Case 2		
	Pre-intervention		Post-intervention	Pre-intervention		Post-intervention	Pre-intervention		Post-intervention	Pre-intervention		Post-intervention
Right vertebral artery luminal diameter (mm)	2.1		2.7	NA		NA	NA		NA	NA		NA
Left vertebral artery luminal diameter (mm)	NA		NA	3.2		3.5	3.0		3.4	3.6		3.8
Vmax (cm/s)	50		74	38		39	35		37	41		38
Ratio of diameter	1.29			1.09			1.06			1.19		
Ratio of blood flow volume	2.77			1.43			1.26			2.01		
NRS dizziness	6	1		10	2		4	2		3	1	
NRS neck pain	4	2		6	1		3	1		2	0	

Blood flow volume is proportional to the fourth power of the radius. Ratio of diameter = diameter (post-intervention)/diameter (pre-intervention of first treatment), ratio of blood flow volume = blood flow volume (post-intervention)/blood flow volume (pre-intervention of first treatment).

NA = not applicable, NRS = numerical rating scale, Vmax = maximum of blood flow velocity.

### 2.2. Case 2

A 49-year-old female patient without previous medical history, presented with a 2-year history of frequent dizziness. She felt head heaviness without any rotational vertigo, and her heaviness lasted for several minutes to hours, which caused her poor sleep quality. She also presented with neck pain, dry eyes, and blurred vision, without symptoms of spontaneous nystagmus, tinnitus or hearing loss, unilateral limb weakness, and dysarthria. On examination, the change in blood pressure measured in the supine and standing position was within normal range, and the patient did not report dizziness when getting up from supine position. The blood pressure was also normal. No nystagmus or dizziness was evoked by the vestibular function testing and cervicoocular reflex. She could maintain a steady gait and keep her balance regardless of having her eyes opened or closed. No definite neurological deficit was observed during clinical evaluation. X-ray examination showed that her cervical spine appears straight, with posterior vertebral osteophytes at C3 to C5. Based on her symptoms, physical, and radiographic examinations, CGD was diagnosed. NRS dizziness and neck pain intensities were 10 and 6, respectively. CVA ultrasound revealed that the luminal diameter of the left VA was 3.2 mm without atherosclerosis (Fig. 1C). She had received manual therapy before, but had no positive clinical effect, so she turned to FSN treatment on September 24, 2021, and completed 3 sessions within 1 month.

**Figure 3.**
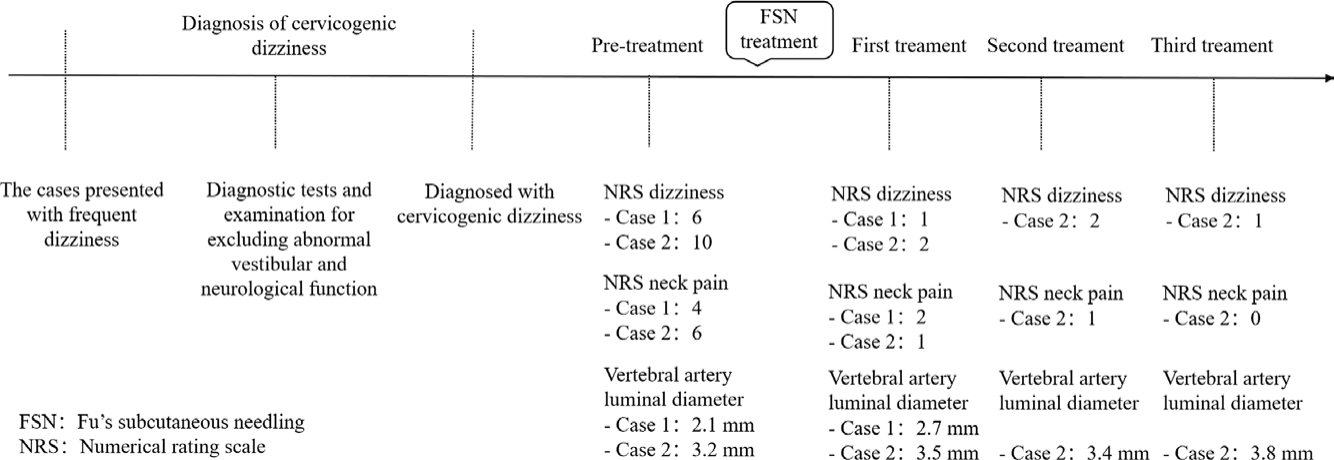
Timeline of FSN treatment with cervicogenic dizziness patients. FSN = Fu's subcutaneous needling, NRS = numerical rating scale.

Immediately after her first FSN treatment, she experienced relief from her neck pain and dizziness. NRS dizziness and neck pain intensities were significantly reduced to 2 and 1 respectively (Table 1). CVA ultrasound instantly showed that the luminal diameter of the left VA was 3.5 mm (Fig. 1D and see Supplemental Video S1, Supplemental Digital Content, http://links.lww.com/MD/I322, which demonstrates the change in diameter), which was 0.3 mm wider than before. After 3 sessions of treatment, the luminal diameter of VA was 3.8 mm, and the patient’s symptoms disappeared almost completely. The timeline of FSN treatment for the 2 CGD patients is shown in Figure 3.

CVA ultrasound is a noninvasive and quick method of assessment, which we used to evaluate cerebral blood flow in these 2 cases.^[10]^ According to the Hagen–Poiseuille equation, an important theoretical basis for the study of circulation and hemotheology, blood flow volume is proportional to the fourth power of the radius.^[11]^ A 1.29-fold increase in diameter (twice the radius) increased the VA blood flow volume by 2.77-fold in Case 1. Similarly, a 1.19-fold increase in diameter increased the VA blood flow volume by 2.01-fold in Case 2, after 3 sessions of treatment (Table 1). We only examined the VAs which enter the vertebrobasilar system and supply blood to the brainstem and cerebellum. Dizziness or ischemic stroke can occur due to insufficient blood supply.^[12]^ Since the VAs merge into the basilar artery, VA enlargement may improve the blood supply to the vertebrobasilar system, and therefore improve dizziness.

## 3. Discussion

CGD accounts for 89% of dizziness according to a large-scale clinical observation.^[13]^ In the absence of a “gold standard” of CGD diagnosis, Li et al proposed a diagnostic criteria for CGD.^[2]^ Nevertheless, pathogenesis of CGD still remains unclear. Proprioceptive and vascular factors are the main etiologies suggested by most recent studies,^[3,14,15]^ both of which we consider to have close relationship with muscles having MTrPs. Pharmacological treatment, physical treatment, acupuncture, and surgical treatment have shown positive effect on CGD.^[16–19]^ Among the conservative non-pharmaceutical treatments, physical treatments focus on reducing muscle stiffness and spasms to relieve neck pain and improve dizziness,^[17]^ while acupuncture emphasized on accelerating BFV with an objective outcome measure of average vertebral-basilar artery BFV in improving dizziness.^[18]^ However, increased BFV cannot reflect the blood flow volume when diameter is unknown. According to the Hagen–Poiseuille equation, an enlargement in diameter increases the blood flow volume. Therefore, our study used CVA ultrasound for the outcome measurement of only the VA luminal diameter, which has never been used before, for as far as we know.

From our study, the VA diameter of both cases enlarged with increased or constant BFV. There is a possible underlying mechanism that subcutaneous stretching enlarges adjacent VA using FSN needle. Stretching subcutaneously may inactivate MTrPs of scalene muscle,^[20]^ which are related to the smooth muscles,^[21]^ the main layer of the artery wall. Another possible mechanism is that subcutaneous stretching may stimulate the vascular endothelial production of nitric oxide, which is one of the main factors for vasodilatation.^[22]^

Due to the multiple collateral circulations of the posterior circulation, short-term decreased perfusion does not form an ischemic area, so the clinical symptoms are not severe at the beginning, and only dizziness may be observed. Identifying stroke in a patient with acute dizziness is challenging particularly in the absence of the accompanying neurological symptoms and signs.^[23]^ A study showed that patients with isolated dizziness and vascular risk factors have a higher prevalence of stroke and hypoperfusion.^[24]^ Although dizziness is not usually a serious problem, it may be a sign of ischemic stroke.^[1]^ Moreover, decreased blood flow volume can cause diameter reduction, which reflects a structural modification of the arterial wall.^[25]^ When decreased blood flow volume occurs over a long time, it may develop into arterial stenosis, which may further reduce blood flow in a significantly narrower VA, and eventually result in signs and symptoms of cerebrovascular diseases, such as transient ischemic attack or stroke.^[26]^ Some studies have provided the evidence that acupuncture increases cerebral blood flow in several cerebrovascular diseases such as post-stroke rehabilitation and Alzheimer disease.^[27,28]^ Thus, this study may provide a safe strategy to relieve CGD and possibly prevent cerebrovascular diseases with increased VA blood flow.

## 4. Conclusion

Subcutaneous stretching provided remarkable outcomes in VA diameter enlargement, and increased blood supply in both case studies. Considering these outcomes, we believe that stretching subcutaneously provides a safe, convenient, and immediate solution, which will help in diagnosing and treating CGD with ultrasound. Eventually, this treatment may benefit ischemic stroke patients, eliminate or reduce patients with disabilities, and help save millions of lives. However, this study observed only 2 cases without long-term follow-up. Further studies including randomized controlled trials with sufficient number of subjects are needed for better evaluation on its clinical efficacy.

## Acknowledgments

We are grateful to the patients for their participation in the study and allowing for publication of these case reports. We thank Dr Alexander B. Mearns, Private Clinic, Tain, Ross-Shire, Scotland, Prof Jian Sun in Guangdong Hospital of Traditional Chinese Medicine, MD, PhD. Xiaolin Yang and Mr. Yuetong Peng in Guangzhou University of Chinese Medicine for their excellent technical assistance, and Bing Wang in Nanjing FSN Institute for producing several of the figures.

## Author contributions

**Conceptualization:** Qingtao He, Zhonghua Fu.

**Investigation:** Qingtao He.

**Methodology:** Qingtao He, Huiyi Huang, Zhonghua Fu.

**Project administration:** Qingtao He, Huiyi Huang, Hongyu Liang.

**Supervision:** Li-Wei Chou, Zhonghua Fu.

**Visualization:** Huiyi Huang, Li-Wei Chou.

**Writing – original draft:** Qingtao He, Huiyi Huang.

**Writing – review & editing:** Li-Wei Chou, Zhonghua Fu.

## Supplementary Material






