# Supplementary material for: Subcutaneous stretching enlarges adjacent vertebral artery instantly in patients with cervicogenic dizziness: Two case reports
Source: Medicine (Baltimore). 2023 Feb 3;102(5):e32643. doi: 10.1097/MD.0000000000032643 (PMC9901990; doi:10.1097/MD.0000000000032643)

**Supplemental Figure.** Figure that illustrates the cervical spine X-ray examination of Case 1. (A) Cervical spine mouth-open view. (B) Cervical spine anteroposterior view. (C) Cervical spine lateral view.

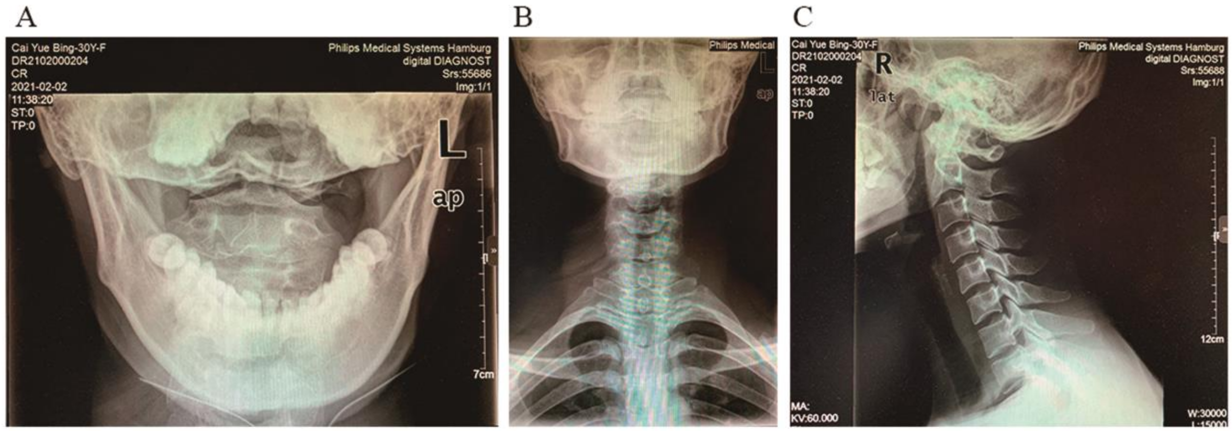

Supplement: Supplementary file 1 [file medi-102-e32643-s001.pdf]
