# Supplementary figures and images for: Subcutaneous stretching enlarges adjacent vertebral artery instantly in patients with cervicogenic dizziness: Two case reports
Source: Medicine (Baltimore). 2023 Feb 3;102(5):e32643. doi: 10.1097/MD.0000000000032643 (PMC9901990; doi:10.1097/MD.0000000000032643)

## Slide 1
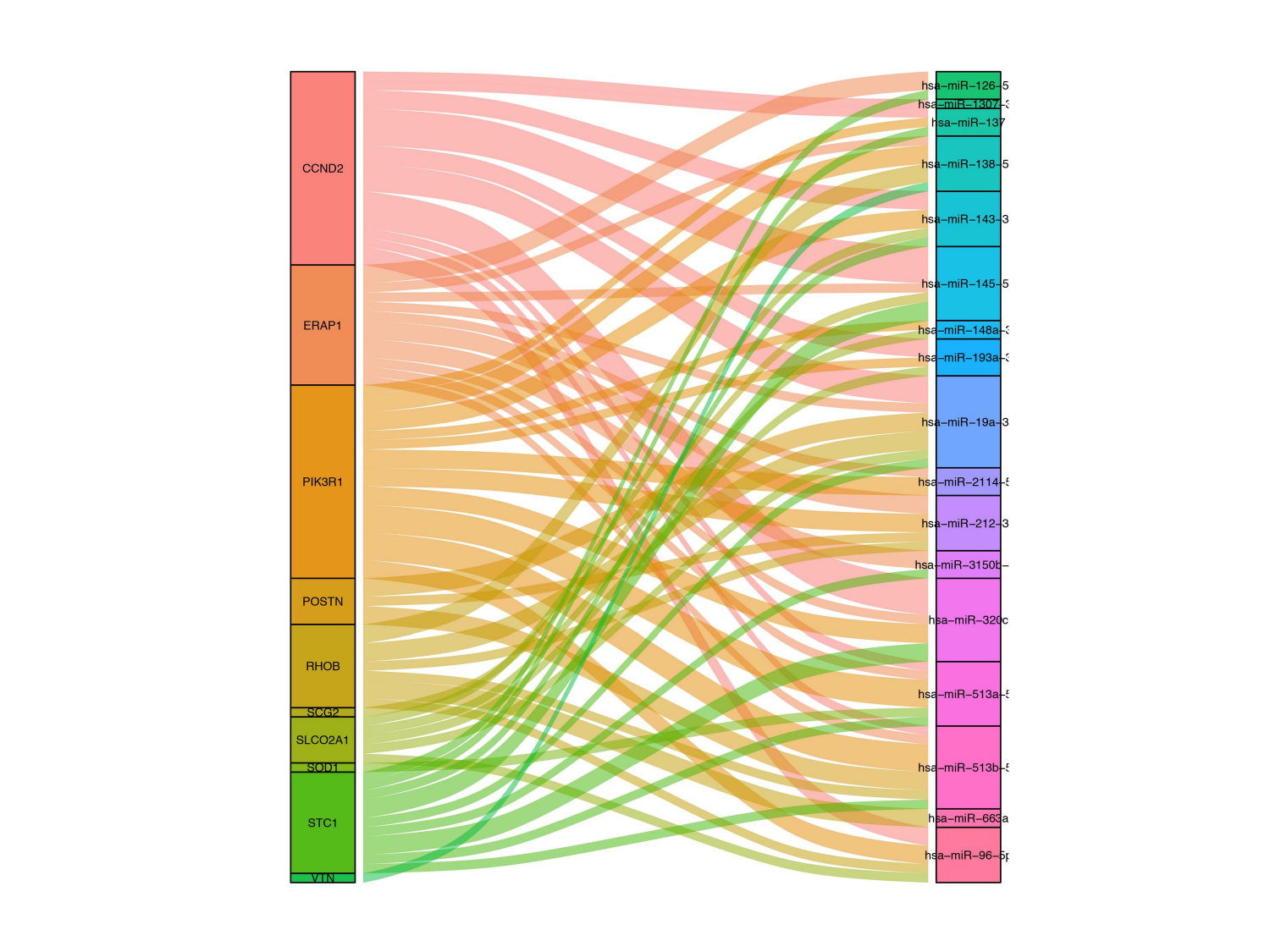

Supplement: Supplementary file 2 [file medi-102-e32643-s002.pptx]
